# Maternal serum levels of C-reactive protein at early pregnancy to predict fetal growth restriction and preterm delivery: A prospective cohort study

**DOI:** 10.18502/ijrm.v18i3.6710

**Published:** 2020-03-29

**Authors:** Roshan Nikbakht, Elham Karimi Moghadam, Zeinab Nasirkhani

**Affiliations:** Fertility Infertility and Perinatology Research Center, Ahvaz Jundishapur University of Medical Sciences, Ahvaz, Iran.

**Keywords:** C-reactive protein, Small for gestational age, Preterm birth.

## Abstract

**Background:**

A considerable evidence suggests that maternal inflammation dysregulation may play as a risk factor for both maternal and neonatal outcomes. **Objective:** The study's objectives were designed to evaluate the correlation between serum C-reactive protein (CRP) levels, as an inflammation factor, preterm delivery, and small for gestational age (SGA) births.

**Materials and Methods:**

This prospective cohort study was conducted on 120 singleton pregnant women with gestational age less than 20 wk. Maternal CRP serum concentration was measured before 20 wk gestation. Patients were followed-up until the delivery and final outcomes of pregnancy were recorded in terms of preterm delivery and SGA births. **Results**: Serum CRP levels in participants with normal fetuses and SGA births were 4.09 ± 1.35 mg/l and 6.04 ± 3.29 mg/l, respectively (p = 0.19), while in cases of preterm delivery, it was 9.63 ± 5.78 mg/l (p < 0.001). By using receiver operating characteristic (ROC) curve, serum CRP levels (cut-off point 5.27 mg/l, area 0.836) had acceptable diagnostic accuracy value in distinguishing preterm delivery (sensitivity (75%), specificity (86.1%), positive predictive value (37.5%), negative predictive value (96.87%), accuracy (85%)) and serum CRP levels (cut-off point 6.67 mg/l, area 0.673) in distinguishing SGA births (sensitivity (50%), specificity (91.2%), positive predictive value (23.07%), and negative predictive value (97.19%), and accuracy (89.16 %)).

**Conclusion:**

Higher maternal serum CRP levels measured early in pregnancy may associate with higher risk of preterm delivery and SGA.

## 1. Introduction 

Preterm delivery is a significant health problem, leading cause of long-term neonatal illness, and is accompanied by behavioral consequences and death in about 75% of survivors (1). Similarly, small for gestational age (SGA) infants are complicated by long-term neurodevelopmental and behavioral problems later in life (2). Despite the severity of these complications, their mechanisms and causes are poorly understood. Hence, there are limitations in the prediction and prevention of preterm delivery and SGA. Most risk factors for SGA focus on abnormalities of the placental–maternal edge. An accumulating body of evidence suggests that these abnormalities mostly originate at early pregnancy and affect implantation and placental development (3, 4). During these processes, immune regulatory changes occur to prevent maternal allogenic response toward the fetus. Few studies have assessed these immune changes due to the lack of maternal blood samples prior to routine prenatal visits. Moreover, Georgiou and colleagues showed that SGA is associated with maternal cytokines abnormalities (4). On the other hand, considerable evidence proposed two important pathways including bacterial infection of the chorioamnion and also intrauterine infections as the significant contributors to preterm delivery (5). These pathways are associated with proinflammatory responses leading to systemic maternal inflammation cytokines production (6). Some studies reported infections as leading causes of preterm deliveries of 30–50% of affected women (7, 8).

C-reactive protein (CRP) is a stable, nonspecific, and sensitive serum marker of systematic inflammation secreted by hepatocytes in response to infection (9). Elevated CRP levels in the peripheral blood circulation are associated with intrauterine infection and amnionitis (10). The increased risk of preterm deliveries with high CRP concentration at early pregnancy is proved by some investigators (11, 12) but not all (13). In addition, there are few studies investigating the association between immune regulatory changes including CRP levels with SGA (2, 4). The early assessment of maternal serum CRP may contribute to early detection of SGA births and to the timely adminstration of preventive procedures. However, a considerable evidence suggests that maternal inflammation dysregulation may play a role for both maternal and neonatal outcomes (2, 3, 14, 15). The paucity of studies has explored the association between maternal serum concentrations of CRP at early pregnancy and preterm delivery and/or neonatal outcomes (2).

The aim of this study was to evaluate the correlation between serum level CRP and maternal and neonatal adverse outcomes including preterm delivery and SGA.

## 2. Materials and Methods 

### Study design and target group 

This prospective cohort study was conducted on 120 pregnant women who delivered at the Obstetrics and Gynecology Department of a tertiary-care hospital affiliated with Ahvaz Jundishapur University of Medical Sciences in the south-west of Iran from January 2016 to March 2017. Pregnant women with a positive chemical test before 20 wk gestation attending for their first routine visit at the prenatal care clinic were recruited in the study. In the first visit, the participants provided the demographic, maternal characteristics, medical history, and maternal urine and blood samples. The maternal age, body mass index (BMI), gravidity were collected from the medical record. The participants were followed-up until delivery to observe the outcomes of the study including preterm and SGA births.

The inclusion criteria for the study consisted of women aged 18-35 yr old, singleton fetus, recalling the exact date of the last menstrual period, GA ≤ 20 wk (5-20 wk of gestation) in the first visit, regular menstrual period, BMI ranging 18-30 kg/m${}^{2}$, and a satisfaction to participate in the study. The exclusion criteria were multiple pregnancies, poor obstetric history, history of preterm labor, history of SGA, failure to recall the exact date of the last menstrual period, patients undergoing hormone therapy, receiving statins, fibrates, and niacin drugs, history of consuming alcohol, drug abuse, medical system disorders such as metabolic syndrome, cardiovascular disorders, hypertension, diabetes (type 1 or 2), autoimmune disorders, inflammation disorders, urinary tract infection, kidney and liver diseases, uterine or cervical abnormality, obese patients with a BMI above 30 kg/m${}^{2}$, any other maternal or fetal complications.

The mean GA at first visit was 16 wk and the range: 5–20 wk. The GA was assigned on the basis of first trimester sonographic findings in which the crown-rump length (CRL) was measured.

The maternal non-fasting blood samples were gathered at the first visit within 10 ml vacutainer tubes at 5–20 gestational wk, and CRP levels were tested along with routine tests. Serum CRP concentrations were determined by high sensitivity enzyme-linked immunosorbent assay (ELISA) (R & D Systems, Minneapolis, MN). The interassay coefficient of variation and detection limit (sensitivity) for CRP were 6.28% and 1ng/ml, respectively.

### Ethical consideration

The study was approved by the Ethical Committee of Ahvaz Jundishapur University of Medical Sciences (Ethical code: IR.AJUMS.REC.1395.547). All participants gave informed consent.

### Statistical analysis

Data were analyzed using SPSS software version 18.0 for windows (Statistical Package for the Social Sciences, SPSS, Chicago, IL, USA). The mean of serum CRP levels was compared between subgroups of variables including maternal age, BMI, gravidity, birth weight, time of delivery, and SGA and non-SGA infants. The means of serum CRP levels between variables were compared using either independent samples *t* test for two groups or one-way ANOVA for more than two groups. ROC curve analysis was utilized to determine the best cut-off point of serum CRP levels for preterm delivery as well as SGA births. The odd ratios and accuracy were calculated to examine the risk of preterm delivery and SGA birth. P-value < 0.05 was considered statistically significant.

## 3. Results 

As shown in Table I, the ranges of participants' age, GA at study assignment, and GA at delivery were 19-35 yr, 5-20 wk, and 31.2-41 wk, respectively. The mean of serum CRP levels of patients was 4.6 ± 2.7 mg/l and the average of infants' birth weight was 3241.7 ± 740.0 gr. Of the 120 pregnant women, the rates of cases with normal fetus, preterm labor and SGA births were 89.16%, 10% and 5%, respectively.

The mean concentration of serum CRP levels was lower but not significant in women with normal fetuses when compared with SGA births' group (p = 0.19). The mean of CRP levels in preterm deliveries was much higher than the term deliveries (p < 0.001) (Table II). Furthermore, the mean of CRP levels significantly increased while the birth weight decreased. We did not find significant differences in the CRP levels between maternal age subgroups (p = 0.14), maternal BMI subgroups (p = 0.40), and gravidity subgroups (p = 0.9) (Table II).

Notably, maternal serum CRP levels exhibited a sensitivity of 75%, the specificity of 86.1%, the accuracy of 85%, and the area under the curve of 0.836 (0.693–0.980) at a cut-off value = 5.27 mg/l as a preterm delivery predictive marker (p < 0.001). The use of a maternal serum CRP level cut-off value of 6.67 mg/l allowed distinguishing SGA and non-SGA births with a sensitivity of 50%, a specificity of 91.2%, an accuracy of 89.2%, area under the curve of 0.673 (0.441–0.905) (p = 0.15) (Tables III and IV).

One-unit increase in serum CRP levels elevated the risk of preterm delivery by 2.3-fold (odds ratio = 2.26, 95% CI: 1.28–3.99, p = 0.005). The CRP level ≥ 5.27 mg/l was significantly associated with a dramatically higher rate of preterm delivery (OR = 18.6, 95% CI: 4.51–76.6, p < 0.0001). Indeed, a one-unit increase in serum CRP levels was not associated with the risk of SGA birth delivery (OR = 1.13, 95% CI: 0.93–1.34, p = 0.20). The CRP level of ≥ 6.67 mg/l was significantly associated with a higher rate of SGA births (OR = 10.4, 95% CI: 1.88–58.4, p = 0.008) (Table V).

**Table 1:** Demographic and maternal characteristics of the study population (n = 120)


	**Mean ± SD**	**Min–Max**
**Age (yr)**	26.5 ± 4.4	19–35
**Gestational age (wk)**	16.1 ± 4.3	5–20
**BMI (kg/m${}^{2}$)**	23.9 ± 3.3	15.6–30
**Gravida (median)**	2	1–5
**Birth weight (gr)**	3241.7 ± 740.0	1500–4600
**C-reactive (mg/l)**	4.6 ± 2.7	1.77–20.20
**Gestational age at delivery (wk)**	37.2 ± 1.7	31.2–41
Yr: Year; wk: Week; BMI: Body mass index; gr: Gram; mg/l (milligram per liter)		

**Table 2:** Early pregnancy maternal serum CRP (mg/l) levels according to maternal characteristics (n = 120)


	**N**	**Mean ± SD**	**P-value**
**Maternal age**	< 25	44	4.14 ± 1.49	0.14${}^{a}$
	25–30	50	5.2 ± 3.8	
	> 30	26	4.27 ± 1.59	
**BMI**	< 20	18	5.4 ± 4.4	0.40${}^{a}$
	20–25	57	4.6 ± 2.6	
	> 25	45	4.4 ± 2.0	
**Gravidity**	1	30	4.8 ± 2.6	0.90${}^{a}$
	2	61	4.5 ± 2.5	
	≥ 3	29	4.7 ± 3.5	
**Birth weight**	< 2000	13	9.3 ± 5.6	< 0.001${}^{a}$
	2000–3500	66	3.9 ± 1.4	
	> 3500	41	4.2 ± 1.2	
**Preterm delivery**	No	108	4.1 ± 1.3	
	Yes	12	9.6 ± 5.8	< 0.001${}^{b}$
**SGA**	No	114	4.5 ± 2.7		
	Yes	6	6.1 ± 3.3	0.20${}^{b}$
${}^{a}$The ANOVA test was used ${}^{b}$The student *t* test was utilized SGA: Small for gestational age; BMI: Body mass index; N: Number; SD: Standard deviation				

**Table 3:** ROC curves for maternal serum CRP levels for predicting preterm delivery and SGA


**Variables**	**Surface under area**	**P-value**	**CI 95%**	
		**Lower limit**	**Upper limit**
**Preterm **	0.836	< 0.01	0.693	0.980
**SGA**	0.673	0.15	0.441	0.905
ROC: Receiver operating characteristic; CRP: C-reactive protein; SGA: Small for gestational age; CI: Confidence interval				

**Table 4:** Accuracy parameters of the study


	**Sensitivity (%)**	**Specificity (%)**	**PPV (%)**	**NPV (%)**	**Accuracy (%)**
**Preterm delivery (CRP ≥ 5.27 mg/l)**	75	86.1	37.5	96.87	85
**SGA (CRP ≥ 6.67 mg/l)**	50	91.2	23.07	97.19	89.2

**Table 5:** Logistic regression analysis for maternal serum CRP levels and risk of Preterm and SGA birth


	**B**	**SE**	**Odds**	**P-value**	**95% CI**
**Preterm delivery**						
	**One-unit increase in CRP levels**	0.89	0.29	2.26	< 0.01	1.28–3.99
	**CRP ≥ 5.27 mg/l**	2.93	0.72	18.6	< 0.01	4.51–76.6
**SGA **						
	**One-unit increase in CRP levels**	0.12	0.09	1.13	0.20	0.93–1.34
	**CRP ≥ 6.67 mg/l**	2.34	0.59	10.4	0.01	1.88–58.4
SE: Standard error; CRP: C-reactive protein; CI: Confidence interval Logistic regression analysis was utilized						

## 4. Discussion 

Our findings showed that maternal serum CRP levels at early gestation are a useful predictor of preterm delivery. This finding is compatible with several other studies that measured the maternal serum CRP levels before 20 wk gestation (1, 8, 12, 16), and those studies measured serum CRP levels after 20 wk, for example, a study by Grgic and colleagues (17), at 20-24 wk gestation, and a study by Shahshahan and colleagues (18), at 24-34 wk of gestation, which reported a significant increase in the preterm group compared to the term deliveries in patients with higher CRP serum levels. However, this is in contrast to studies reporting no significant difference in serum CRP levels measured at 15-18 wk gestation (9, 13, 19) and at 16-27 wk of gestation published by Bullen and colleagues (20) between the preterm and term deliveries. Bullen and colleagues demonstrated that CRP levels were non-significantly elevated for preterm (5.5 µg/ml) versus term deliveries (4.8 µg/ml) and were higher in the preterm deliveries with histologic chorioamnionitis (6.3 µg/ml). They also showed a significant association between preterm and CRP only in higher pregestational BMI (> 30) women (7.5 mg in preterm vs 6.6 mg/ml in term deliveries; p < 0.05) (20). Moreover, we found that the best cut-off value of maternal serum CRP levels in the first trimester was 5.27 mg/l for predicting the preterm delivery. This cut-off differs between literature; Grgic and colleagues (17) described a cut-off of 4 mg/l, Pitiphat and colleagues reported a CRP serum levels ≥ 8 mg/L (21), and Ertas and colleagues (22) described a CRP cut-off of 9.66 mg/L with a greater risk of preterm delivery. The potential explanations depend on many factors such as the inclusion and exclusion criteria of the study populations and mainly a time period of measurement. The increase in maternal serum CRP in preterm deliveries reflects an activated inflammatory pattern as a result of upregulated secretion of cytokines (15).

As observed through the literature review, there are some studies showed a significant association between CRP at early pregnancy and preterm delivery (1, 8, 12, 16), while others failed to confirm it (9, 13, 19). Our finding that maternal serum levels of CRP at 5-20 wk are associated with preterm delivery suggests that inflammatory process can be apparent from early pregnancy. We showed the OR of 1.31 (CI 95%: 0.93–1.34, p = 0.20) in association between one-unit increase in serum CRP levels and SGA births, but this increased dramatically (the OR of 10.4, CI 95%: 1.85–58.47, p <0.01) in association with CRP levels above 6.67 mg/l with SGA births. Moreover, in our study, the mean CRP levels did not differ significantly between women with SGA and those with non-SGA births, however, this comparison was significant when we compared the categories of birth weight; we reported significantly higher CRP serum levels at early pregnancy in women with neonate's birth weight < 2000 gr.

Previous studies on biomarker of SGA have assayed blood samples collected during pregnancy, however, most of these studies addressed abnormalities in maternal–fetal interface immune regulation, there are considerable inconsistencies between them, which can be attributed to varying time window for blood collection and racial differences (1, 4, 23-26). In a study by Vecchie and colleagues in 2018, it has been reported that CRP serum levels at midpregnancy were significantly higher in the group with maternal adverse outcomes and these mothers have smaller babies (15).

## 5. Conclusion 

In conclusion, we observed a positive association between serum CRP levels and higher rates of preterm deliveries and SGA births. The novelty of our study was measuring CRP levels in early pregnancy and also assessing the preterm deliveries and SGA birth together. Hence, the current study can add some piece of information on the existing knowledge in relation between early CRP levels and preterm deliveries and SGA birth. However, the number of cases to draw a firm conclusion from is also insufficient. Moreover, the range to measure CRP has been too wide which might reduce the significance of the study and produce biasness. Another limitation that needs to be mentioned is that CRP itself is a very nonspecific marker which in spite of using strict exclusion criteria can be positive or high for other reasons that can produce some biasness. Finally, higher maternal serum CRP levels measured early in pregnancy may associate with higher risk of preterm delivery and SGA.

##  Conflicts of Interest 

The authors declare that there is no conflict of interest.

## References

[1] Ferguson Kelly K., Mcelrath Thomas F., Chen Yin-Hsiu, Mukherjee Bhramar, Meeker John D. (2014). Longitudinal Profiling of Inflammatory Cytokines and C-reactive Protein during Uncomplicated and Preterm Pregnancy. American Journal of Reproductive Immunology.

[2] Pearce Brad D., Nguyen Phuong H., Gonzalez-Casanova Ines, Qian Yuchen, Omer Saad B., Martorell Reynaldo, Ramakrishnan Usha (2016). Pre-pregnancy maternal plasma cytokine levels and risks of small-for-gestational-age at birth. The Journal of Maternal-Fetal & Neonatal Medicine.

[3] Ferguson Kelly K., Kamai Elizabeth M., Cantonwine David E., Mukherjee Bhramar, Meeker John D., Mcelrath Thomas F. (2018). Associations between repeated ultrasound measures of fetal growth and biomarkers of maternal oxidative stress and inflammation in pregnancy. American Journal of Reproductive Immunology.

[4] Georgiou Harry M., Thio Yulinda S., Russell Chris, Permezel Michael, Heng Yujing J., Lee Stephen, Tong Stephen (2011). Association between maternal serum cytokine profiles at 7-10 weeks' gestation and birthweight in small for gestational age infants. American Journal of Obstetrics and Gynecology.

[5] Park Hyunsoo, Park Kyo Hoon, Kim Yu Mi, Kook Song Yi, Jeon Se Jeong, Yoo Ha-Na (2018). Plasma inflammatory and immune proteins as predictors of intra-amniotic infection and spontaneous preterm delivery in women with preterm labor: a retrospective study. BMC Pregnancy and Childbirth.

[6] Ryu Hyun Kyung, Moon Jong Ho, Heo Hyun Ji, Kim Jong Woon, Kim Yoon Ha (2016). Maternal c-reactive protein and oxidative stress markers as predictors of delivery latency in patients experiencing preterm premature rupture of membranes. International Journal of Gynecology & Obstetrics.

[7] R Alijahan, S Hazrati, M Mirzarahimi, F Pourfarzi, P. Ahmadi Hadi

[8] Darling Anne Marie, Mcdonald Chloe R., Conroy Andrea L., Hayford Kyla T., Liles W. Conrad, Wang Molin, Aboud Said, Urassa Willy S., Kain Kevin C., Fawzi Wafaie W. (2014). Angiogenic and inflammatory biomarkers in midpregnancy and small-for-gestational-age outcomes in Tanzania. American Journal of Obstetrics and Gynecology.

[9] Bakalis Spyros P., Poon Leona C. Y., Vayna Anna-Maria, Pafilis Ioannis, Nicolaides Kypros H. (2012). C-reactive protein at 11–13 weeks’ gestation in spontaneous early preterm delivery. The Journal of Maternal-Fetal & Neonatal Medicine.

[10] Musilova Ivana, Kacerovsky Marian, Stepan Martin, Bestvina Tomas, Pliskova Lenka, Zednikova Barbora, Jacobsson Bo (2017). Maternal serum C-reactive protein concentration and intra-amniotic inflammation in women with preterm prelabor rupture of membranes. PLOS ONE.

[11] Cotechini Tiziana, Komisarenko Maria, Sperou Arissa, Macdonald-Goodfellow Shannyn, Adams Michael A., Graham Charles H. (2014). Inflammation in rat pregnancy inhibits spiral artery remodeling leading to fetal growth restriction and features of preeclampsia. The Journal of Experimental Medicine.

[12] Kumarathasan P., Vincent R., Das D., Mohottalage S., Blais E., Blank K., Karthikeyan S., Vuong N.Q., Arbuckle T.E., Fraser W.D. (2014). Applicability of a high-throughput shotgun plasma protein screening approach in understanding maternal biological pathways relevant to infant birth weight outcome. Journal of Proteomics.

[13] Ghezzi Fabio, Franchi Massimo, Raio Luigi, Di Naro Edoardo, Bossi Giuseppina, D'eril Gian Vico Melzi, Bolis Pierfrancesco (2002). Elevated amniotic fluid C-reactive protein at the time of genetic amniocentesis is a marker for preterm delivery. American Journal of Obstetrics and Gynecology.

[14] Garcia-Flores Valeria, Romero Roberto, Miller Derek, Xu Yi, Done Bogdan, Veerapaneni Chharitha, Leng Yaozhu, Arenas-Hernandez Marcia, Khan Nabila, Panaitescu Bogdan, Hassan Sonia S., Alvarez-Salas Luis Marat, Gomez-Lopez Nardhy (2018). Inflammation-Induced Adverse Pregnancy and Neonatal Outcomes Can Be Improved by the Immunomodulatory Peptide Exendin-4. Frontiers in Immunology.

[15] Ba Vecchié A, F Carbone, D Maggi, A Ferraiolo, B Carloni, .

[16] Hvilsom Gitte, Thorsen Poul, Jeune Bernard, Bakketeig Leiv (2002). C-reactive protein: a serological marker for preterm delivery?. Acta Obstetricia et Gynecologica Scandinavica.

[17] G Grgic, F Skokic, G Bogdanovic

[18] Shahshahan Zahra, Rasouli Ousha (2014). The use of maternal C-reactive protein in the predicting of preterm labor and tocolytic therapy in preterm labor women. Advanced Biomedical Research.

[19] Ozer Kamer T., Kavak Zehra N., Gökaslan Hüsnü, Elter Koray, Pekin Tanju (2005). Predictive power of maternal serum and amniotic fluid CRP and PAPP-A concentrations at the time of genetic amniocentesis for the preterm delivery. European Journal of Obstetrics & Gynecology and Reproductive Biology.

[20] Bl Bullen, Nm Jones, Cb Holzman, Y Tian, Pk Senagore, P Thorsen

[21] Pitiphat Waranuch, Gillman Matthew W., Joshipura Kaumudi J., Williams Paige L., Douglass Chester W., Rich-Edwards Janet W. (2005). Plasma C-Reactive Protein in Early Pregnancy and Preterm Delivery. American Journal of Epidemiology.

[22] Ertas Ibrahim E., Kahyaoglu Serkan, Yilmaz Bulent, Ozel Murat, Sut Necdet, Guven Melih A., Danisman Nuri (2010). Association of maternal serum high sensitive C-reactive protein level with body mass index and severity of pre-eclampsia at third trimester. Journal of Obstetrics and Gynaecology Research.

[23] Briana Despina D., Liosi Sofia, Gourgiotis Dimitrios, Boutsikou Maria, Marmarinos Antonios, Baka Stavroula, Hassiakos Dimitrios, Malamitsi-Puchner Ariadne (2012). Fetal concentrations of the growth factors TGF-α and TGF-β1 in relation to normal and restricted fetal growth at term. Cytokine.

[24] Conde-Agudelo A, Papageorghiou At, Kennedy Sh, Villar J (2013). Novel biomarkers for predicting intrauterine growth restriction: a systematic review and meta-analysis. BJOG: An International Journal of Obstetrics & Gynaecology.

[25] Ernst Gesina D.S., De Jonge Layla L., Hofman Albert, Lindemans Jan, Russcher Henk, Steegers Eric A.P., Jaddoe Vincent W.V. (2011). C-reactive protein levels in early pregnancy, fetal growth patterns, and the risk for neonatal complications: the Generation R Study. American Journal of Obstetrics and Gynecology.

[26] Lindner U., Tutdibi E., Binot S., Monz D., Hilgendorff A., Gortner L. (2013). Levels of Cytokines in Umbilical Cord Blood in Small for Gestational Age Preterm Infants. Klinische Pädiatrie.

